# Insights into the Stress Response Triggered by Kasugamycin in *Escherichia coli*

**DOI:** 10.3390/antibiotics5020019

**Published:** 2016-06-01

**Authors:** Christian Müller, Lena Sokol, Oliver Vesper, Martina Sauert, Isabella Moll

**Affiliations:** Max F. Perutz Laboratories, Center for Molecular Biology, Department of Microbiology, Immunobiology and Genetics, University of Vienna, Vienna Biocenter (VBC), Dr. Bohr-Gasse 9/4, A-1030 Vienna, Austria; christian_mueller@univie.ac.at (C.M.); lena.sokol@pathology.unibe.ch (L.S.); oliver.vesper@imba.oeaw.ac.at (O.V.); martina.sauert@univie.ac.at (M.S.)

**Keywords:** kasugamycin, translation initiation, leaderless mRNA, *Escherichia coli*

## Abstract

The bacteriostatic aminoglycoside antibiotic kasugamycin inhibits protein synthesis at an initial step without affecting translation elongation. It binds to the mRNA track of the ribosome and prevents formation of the translation initiation complex on canonical mRNAs. In contrast, translation of leaderless mRNAs continues in the presence of the drug *in vivo.* Previously, we have shown that kasugamycin treatment in *E. coli* stimulates the formation of protein-depleted ribosomes that are selective for leaderless mRNAs. Here, we provide evidence that prolonged kasugamycin treatment leads to selective synthesis of specific proteins. Our studies indicate that leaderless and short-leadered mRNAs are generated by different molecular mechanisms including alternative transcription and RNA processing. Moreover, we provide evidence for ribosome heterogeneity in response to kasugamycin treatment by alteration of the modification status of the stalk proteins bL7/L12.

## 1. Introduction

The bactericidal aminoglycoside antibiotic kasugamycin (Ksg) inhibits protein synthesis in eukaryotic and prokaryotic cells at an initial step [1] without affecting translational elongation [2]. Several experiments have suggested that it prevents the formation of the pre-initiation complex by interfering with the binding of the initiator fMet-tRNA^fMet^ to the ribosomal P-site in *E. coli* ribosomes [3,4]. Structural analyses revealed that the antibiotic binds to the mRNA track of the ribosome, thereby preventing formation of the translation initiation complex [5,6]. Resistance to Ksg is caused by the lack of dimethylation of two adjacent adenosines 1518/1519 in helix 45 at the 3′-end of the 16S rRNA [7]. Intriguingly, this post-transcriptional modification is the only universally conserved modification common to the rRNA of prokaryotes and eukaryotes [8], suggesting an important function that still remains to be elucidated. Nevertheless, structural analyses indicate that the modification alters the conformation and flexibility of the ultimate stem-loop in the 16S rRNA [9] and, thus, the dimethylated adenosine residues might assist in correct folding of the 3′-terminus of the 16S rRNA to ensure the availability of the anti-Shine-Dalgarno (aSD) sequence required for translation initiation on canonical transcripts [10].

At variance with the characterized effect of the antibiotic Ksg on the initiation of protein synthesis on canonical transcripts, translation of leaderless mRNAs (lmRNAs) continues in the presence of the drug *in vivo* [1,11]. Despite the fact that the conserved nucleotides A1518 and A1519 of 16S rRNA, the methylation of which causes sensitivity to the drug, lie in close proximity to the aSD sequence, Ksg does not interfere with formation of the SD-aSD helix during ternary complex formation [12]. In addition, the activity of initiation factor 3 (IF3) was not affected by Ksg [12], even though IF3 interacts with the ribosome close to the Ksg binding site [13]. However, Ksg differentially affects the formation of 30S and 70S initiation complexes [3,12] underpinning the 70S initiation pathway for lmRNAs [14]. Surprisingly, ribosome profile analyses revealed the appearance of a ribosomal subpopulation after Ksg treatment in *E. coli*, which sediments at 61S [15]. These ribosomes contain a normal 50S subunit but a 30S species with a reduced protein complement: at least six proteins are lacking from the 30S subunit, among which are the functionally important proteins bS1 and uS2, both of which are essential for translation of canonical mRNAs [16,17,18,19], but are dispensable for translation of lmRNAs [15,17,18,19]. The characterization of the 61S particles revealed that they form initiation complexes on lmRNA comparable to 70S ribosomes. However, in contrast to 70S ribosomes these 61S particles are competent in translation of lmRNA in the presence of Ksg *in vivo* and *in vitro* [15].

Treatment of *E. coli* with antibiotics that inhibit transcription or translation triggers the activation of the *mazEF* module, a so-called type 2 toxin-antitoxin (TA) system [20,21]. In general, type 2 TA systems comprise two transcriptionally and translationally coupled genes that encode for a labile antitoxin and a stable toxin. As the labile antitoxin interacts with the toxin, the toxic activity is inhibited. However, during stress conditions, which inhibit the continuous expression of the TA genes, degradation of the antitoxin releases the active toxin that interferes with diverse activities in the cell like replication, transcription, or translation [22]. The toxin MazF is an endoribonuclease that degrades the majority of bulk mRNA by cleaving at single-stranded ACA sites [23]. In addition, its activation results in the processing of distinct transcripts within their 5′-UTR, which enables their selective synthesis by specialized ribosomes that are likewise modified by the MazF activity [24]. Here, the MazF toxin cleaves the 16S rRNA in the context of mature and active ribosomes close to the 3′-terminus, thereby removing the last 43 nucleotides comprising helix 45 and the aSD sequence (from here on referred to as RNA43). Taken together, these particular processing events performed by the activation of a single endoribonuclease result in a fast and energy-saving reprogramming of the translatome in response to diverse stress conditions [25,26].

Given that the addition of the aminoglycoside Ksg mimics the effect of the toxin MazF in rendering the translational machinery specific for lmRNAs, our observation that several proteins are selectively synthesized in the presence of the antibiotic tempted us to speculate that Ksg treatment triggers the *mazEF* system. We suspected that the MazF toxin is involved in the formation of 5′-terminally processed transcripts that are consequently translated by the 61S ribosomes in the presence of Ksg. Thus, we studied the consequences of Ksg treatment on the transcriptome and translatome in *E. coli*. Our results show that besides formation of protein depleted 61S ribosomes, extended Ksg treatment leads to selective protein synthesis that can be attributed to the formation of leaderless and short-leadered transcripts. Surprisingly, Ksg does not lead to a strong activation of the *mazEF* TA system but our results suggest that the majority of transcripts with short leaders are rather engendered by alternative transcription. In addition, we observed ribosome heterogeneity in response to kasugamycin treatment that is based on the modification status of the stalk proteins bL7/L12.

## 2. Results

### 2.1. Selective Protein Synthesis after Prolonged Kasugamycin Treatment

**Figure 1:**
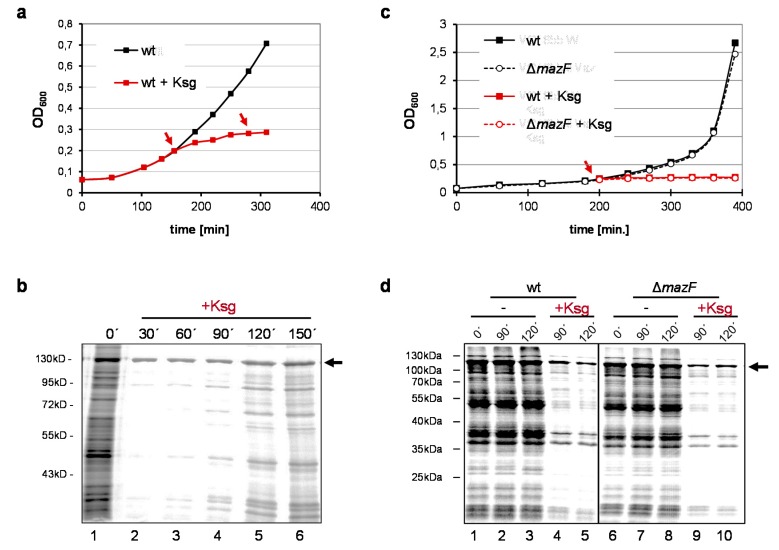
Prolonged Ksg treatment triggers selective protein synthesis. (**a**) Growth of strain MG1655 harboring plasmid pRB381-1, encoding the leaderless *cI-lacZ* fusion gene in the absence (black) and presence of 0.5 mg/ml Ksg (red line) was monitored by measuring the optical density at 600 nm. Addition of Ksg is indicated by red arrows. Error bars representing the standard deviation of triplicate samples are hidden by the symbols. (**b**) At indicated time points before and after addition of Ksg pulse labeling was performed and the labelled proteins were separated by SDS-PAGE. The position of the CI-LacZ fusion protein is indicated by an arrow. (**c**) Growth of strain MG1655 (solid line) in the absence (black) and presence of 0.5 mg/ml Ksg (red line) was compared to growth of strain MG1655Δ*mazF* (dotted line) both harboring plasmid pRB381-1. Addition of Ksg is indicated by a red arrow. Again, error bars representing the standard deviation of triplicate samples are hidden by the symbols. (**d**) At time points indicated pulse labeling was performed and the labelled proteins were separated by SDS-PAGE. The arrow indicates the CI-LacZ fusion protein.

As shown before, the addition of Ksg results in the cessation of *E. coli* growth (Figure 1a) and the inhibition of protein synthesis (Figure 1b, lanes 2–3). However, pulse labeling experiments employing *E. coli* strain MG1655 harboring plasmid pRB381-1 [14], encoding the leaderless *cI-lacZ* fusion gene, revealed that translation of a leaderless reporter continued in the presence of 0.5 mg/mL Ksg (Figure 1b, lanes 2–6; [15]). Intriguingly, 90–120 min after addition of the antibiotic, we observed restored *de novo* translation of particular proteins (Figure 1b, lanes 4–6), which was resistant to Ksg even after retreatment of the cells with the antibiotic at time point 120 min (Figure 1b, lane 6). Considering the selective synthesis of proteins encoded by lmRNAs after induction of *mazF* together with the fact that antibiotic treatment can trigger the *mazEF* system [20,21] we hypothesized that Ksg treatment likewise results in activation of MazF. This would in turn lead to the formation of lmRNAs, which are selectively translated in the presence of Ksg. To test for this hypothesis we repeated the growth analysis using the isogenic *mazF* deletion mutant and performed pulse labeling upon addition of Ksg (Figure 1c,d). Surprisingly, the experiment revealed that despite the lack of MazF the same proteins are selectively synthesized after Ksg treatment (Figure 1d, lanes 4–5 and 9–10). This result indicates that MazF is not required for the specific protein synthesis in the presence of Ksg.

### 2.2. Identification of Proteins Selectively Synthesized in the Presence of Kasugamycin

**Figure 2:**
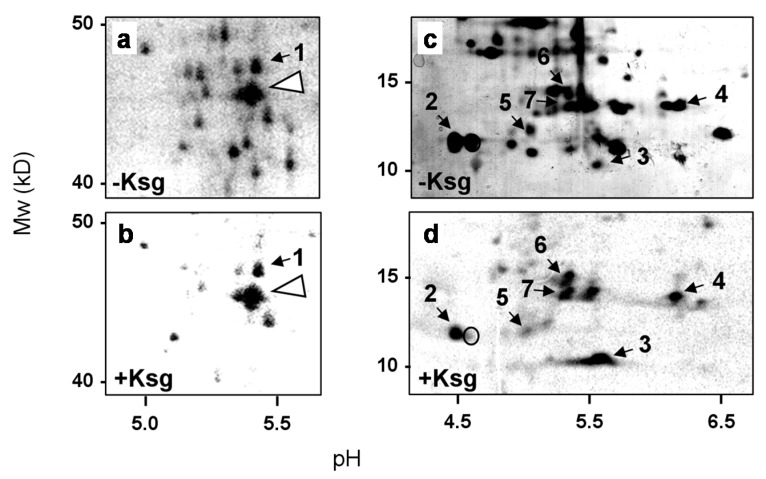
2D gel analysis performed to identify selectively synthesized proteins in the presence of Ksg. (**a**) and (**c**) Sections of the 2D gel performed with samples withdrawn from the untreated strain; (**b**) and (**d**) the same sections of the 2D gel used for the separation of the proteins labeled in the presence of Ksg. The molecular weight is given to the left and the pH values are indicated at the bottom. The proteins identified are numbered according to Table 1. The position of elongation factor 2 is indicated by an arrow head in (**a**) and (**b**) and the position of the unmodified protein uL12 is circled in (**d**).

In *E. coli* only three natural lmRNAs were hitherto known [25,27]. Thus, our observation that the synthesis of several proteins continues in the presence of Ksg even in the absence of *mazF* (Figure 1d) suggests alternative mechanisms involved in the formation of lmRNAs. To this end we first aimed to identify proteins synthesized in the presence of Ksg. Upon pulse labeling the sample, withdrawn 120 min after addition of the antibiotic to the strain MG1655 (Figure 1a), was separated by 2D gel electrophoresis as specified in Materials and Methods and compared to the protein pattern obtained without Ksg treatment (Figure 2). The spots corresponding to selectively synthesized proteins were cut from the gel and identified by mass spectrometry. As shown in Table 1, we identified stress response proteins encoding heat shock as well as cold shock proteins, ribosomal proteins (r-proteins), and ribosome-modifying enzymes. As expected and in contrast to their translatability in the presence of Ksg *in vivo*, these mRNAs are reported to contain long 5′-UTRs or are even located within an operon, like the *rplL* and *dnaJ* mRNAs, encoding r-protein bL7/L12, and the heat-shock chaperone DnaJ (Table 1). In addition, we observed the synthesis of the enzyme RimL, the enzyme acetylating the *N*-terminal serine of the stalk r-protein bL12 to form bL7. Consistent with this observation, in the presence of Ksg we only observed the spot corresponding to the modified r-protein bL7 (Figure 2d, spot #2). In contrast, the signal corresponding to the unmodified r-protein bL12 was not detectable (Figure 2d, open circle). In line with the selective translation of the respective mRNAs even in the *mazF* deletion strain (Figure 1d, lanes 9–10), only a minor fraction of the transcripts have been identified as MazF-targets [25].

### 2.3. Formation of Short-leadered and lmRNAs upon Ksg Treatment

**Figure 3:**
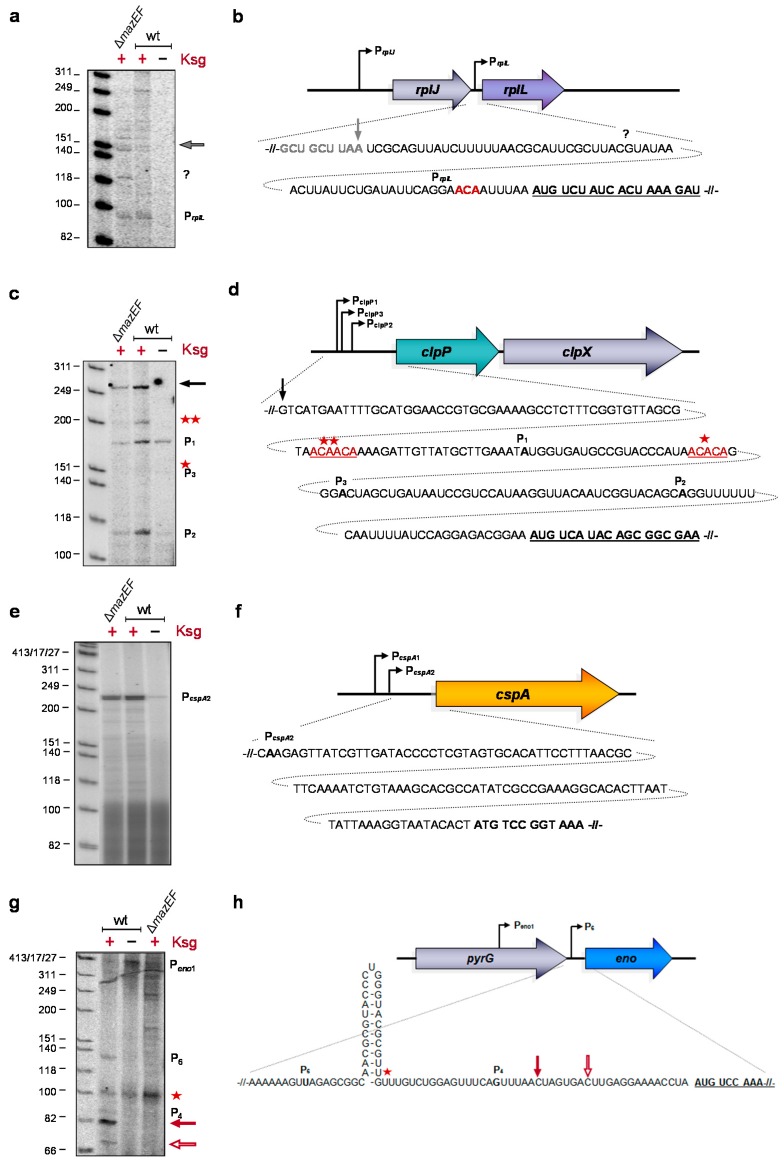
Determination of the 5′-terminus of the *rplL* (**a** and **b**), *clpP* (**c** and **d**), *cspA* (**e** and **f**), and *eno* (**g** and **h**) mRNAs by primer extension analysis. Total RNA was prepared from strain MG1655 before and 120 min after addition of Ksg. Likewise, total RNA was prepared from strain MG1655Δ*mazF* after treatment with Ksg for 120 min and used for the analysis. The analyses were performed in triplicate and one representative autoradiograph is shown. (**b**, **d**, **f**, and **g**) Schematic depictions of promoter positions and sequence of 5′-UTRs and proximal coding regions of the different RNAs are shown. Signals corresponding to transcriptional start sites and processing sites are indicted to the right of the primer extension analysis and in the depiction of the sequence. The lengths of the standard marker are given to the left.

Given that translation of lmRNAs selectively continues in the presence of Ksg [1], the constant synthesis of the respective proteins indicates the leaderless nature of the transcript variants upon Ksg treatment *in vivo*, *i.e.*, under stress conditions induced by the antibiotic. To determine the 5′-termini of these transcripts after Ksg treatment, total RNA was isolated from *E. coli* MG1655 cells grown either in the absence of Ksg or 120 min after addition of the antibiotic or from the isogenic *mazF* deletion strain 120 min after addition of Ksg. The 5′-termini of four selected mRNAs were determined by primer extension analysis, employing primers that bind 50–100 nucleotides downstream of the AUG start codon of the respective transcripts (Table S2). The selected mRNAs encode (i) the r-protein bL7/L12; and (ii) the proteolytic subunit of the ATP-dependent ClpAP serine protease ClpP, as both the *rplL* and *cplP* mRNAs were already identified as the MazF target [25]; (iii) the cold shock protein A [28]; and (iv) the phosphoprotein enolase, which besides catalyzing the interconversion of 2-phosphoglycerate and phosphoenolpyruvate during glycolysis and gluconeogenesis is also a component of the degradosome [29]. As shown in Figure 3c,e,g, primer extension analysis using total RNA purified from untreated cells with primers specific for *clpP, cspA*, and *eno* mRNAs yielded signals that correspond to the transcriptional start at the determined promoters. As the main promoter responsible for transcription of the *rplL* gene is located upstream of the preceding *rplJ* gene, we were not able to determine the corresponding signal by primer extension analysis (Figure 3a). In contrast, employing total RNA purified from Ksg-treated cells, primer extension yielded signals that correspond to alternative lengths of the respective 5′-UTRs (Figure 3a,c,e,g). Using primers specific for the *rplL*, *clpP*, and *cspA* mRNAs, we obtained the same signals employing total RNA purified from both the wild-type strain and the *mazF* deletion strain upon addition of Ksg, supporting the hypothesis that Ksg-dependent selective translation is independent of the activation of MazF. The analysis shown in Figure 3a indicates that after Ksg treatment a weak signal appeared that corresponds to alternative transcription at promoter P*_rplL_* located between the *rplJ* and *rplL* genes [30]. This promoter is localized closely to an ACA site upstream of the AUG start codon, which was identified as a target for MazF cleavage [25]. However, as the signal is likewise present in the *mazF* deletion strain the results suggests that alternative transcription at the P*_rplL_* promoter rather than MazF cleavage is responsible for the formation of an *rplL* mRNA with short leader (Figure 3a,b). Likewise, primer extension analysis using the primer specific for the *clpP* mRNA shows two additional stop signals indicating alternative transcription starting at the P*_clpP_*_2_ promoter 31 nucleotides upstream of the start codon (Figure 3c). Notably, in the wild-type strain, a signal corresponding to MazF cleavage at an ACAACA site within the 5′-UTR appeared after addition of Ksg, which was not visible in the *mazF* deletion mutant (Figure 3c, red stars). However, we did not observe the signal corresponding to the previously reported MazF cutting site at the ACACA site (Figure 3c, red star; [25]). In addition we observed a strong signal that corresponds to transcription starting approximately 180 nucleotides upstream of the start codon (Figure 3c, black arrow). However, hitherto no promoter has been predicted at this position. Taken together, these results indicate that Ksg treatment results mainly in transcription of the respective mRNAs from alternative promoters that are generally located close to the AUG start codon and only a minor fraction of the transcripts is processed by MazF. However, we cannot exclude a processing event by other endoribonucleases besides MazF.

Surprisingly, the analysis performed on the *cspA* mRNA revealed a strong induction of the transcription at the promoter P*_cspA2_* after Ksg treatment of both the wild type and the *mazF* deletion mutant strain (Figure 3e,f). This result is in striking contrast with previous studies that did not reveal the induction of *cspA* expression by Ksg [31].

Primer extension reactions using a primer specific for the *eno* mRNA yielded a faint signal that corresponds to the alternative transcription start P*_eno6_* 76 nucleotides upstream of the *eno* gene in the presence of Ksg (Figure 3g,h, [32]). In addition, we observed two distinct signals that correspond to 5′-UTRs with a length of 16 and 23 nucleotides, respectively, indicating alternative 5′-ends of the mRNA (Figure 3g,h, open and closed red arrow, respectively). Surprisingly, these signals correspond to ACU sites located upstream of the AUG start codon of the *eno* mRNA (Figure 3h, open and closed red arrow), suggesting cleavage by the toxin ChpB, a MazF homolog that specifically cleaves at ACY sequences (Y is U, A, or G) [33]. Moreover, RNA sequencing analysis performed after induction of *mazF* expression reveals that the *eno* mRNA is not processed by MazF [25]. However, this analysis indicates a striking difference between the wild type and the *mazF* deletion mutant strain, where we did not observe these signals. Thus, these results suggest a potential cross activation of the ChpB toxin by MazF, which remains to be elucidated. Interestingly, the primer extension analysis also indicates the presence of a hairpin structure that is removed by the processing event (Figure 3g,h, red star).

Taken together, these results suggest that Ksg treatment induces the generation of variants of specific mRNAs with short leaders by diverse molecular mechanisms, including alternative transcription and mRNA processing. However, the common denominator of these mechanisms is the shortening of the 5′-UTR, including the removal of secondary structures, as shown for the *eno* mRNA (Figure 3g,h).

### 2.4. Selective Translation of mRNAs with Short Leaders upon Kasugamycin Treatment

**Figure 4:**
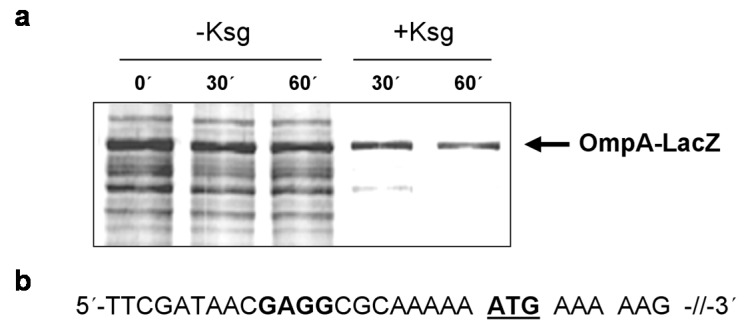
Selective translation of the short-leadered *ompAΔ117*-*lacZ* mRNA in the presence of Ksg *in vivo*. (**a**) Pulse labeling of strain MG1655 harboring plasmid pIM17 grown in the absence or in the presence of Ksg. The arrow indicates the position of the fusion protein. (**b**) The sequence of the 5′-UTR and the proximal coding region of the mRNA is given. The AUG start codon is underlined and the SD sequence is given in bold.

Intriguingly, all tested mRNAs still contain a short 5′-UTR. We therefore asked whether these mRNAs are still translated in the presence of Ksg. For this purpose, *E. coli* strain MG1655 harboring plasmid pIM17, encoding the *ompA*Δ117-*lacZ* fusion gene containing a 5′-UTR of 21 nucleotides in length (Figure 4b), was grown in M9 minimal medium and pulse labeling experiments were performed without antibiotic treatment or 30 or 60 min after addition of 0.5 mg/mL Ksg, respectively. As shown in Figure 4a, translation of the *ompA*Δ117-*lacZ* fusion mRNA prevailed in the presence of the antibiotic comparable to the leaderless *cI-lacZ* mRNA. This result indicates that mRNAs comprising a short unstructured leader are likewise translated in the presence of the translation initiation inhibitor Ksg, reminiscent to the selective translation by MazF-processed 70S^Δ43^ ribosomes that lack the aSD sequence [25].

### 2.5. Formation of RNA43 upon Kasugamycin Treatment

**Figure 5:**
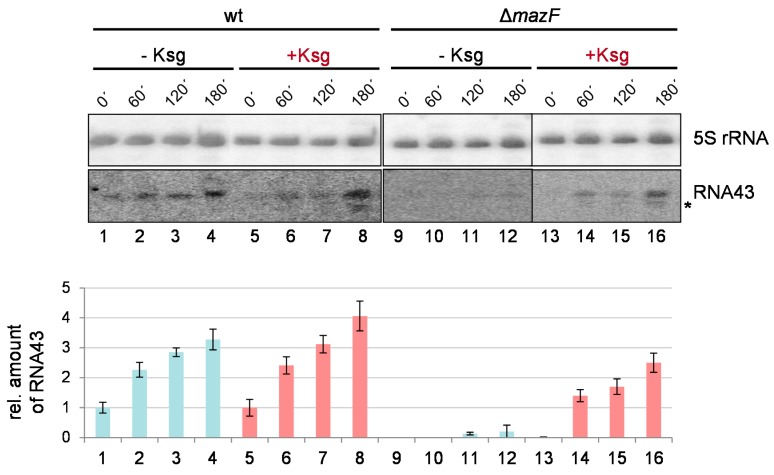
Formation of RNA43 upon Ksg treatment. Total RNA purified from strain MG1655 (lanes 1–8) and MG1655Δ*mazF* (lanes 9–16) at indicated time points without (lanes 1–4 and 9–12) or with the addition of Ksg (lanes 5–8 and 13–16) was subjected to northern blot analysis using probe V7, specific for the RNA43. A probe specific for the 5S rRNA was used as an internal control. The second signal observed after addition of Ksg is marked by an asterisk. The obtained RNA43 signal intensities were quantified and normalized using the 5S rRNA signals. In the graph below, the corresponding relative amounts of RNA43 are given Light blue and red bars indicate without or with Ksg treatment, respectively. The value obtained in strain MG1655 at time point 0’ (lane 1) was set to 1. The error bars represent the standard deviation of triplicate samples.

Taken together, the data shown in Figure 3 do not suggest that Ksg treatment strongly triggers the activation of the *mazEF* TA-system. To further scrutinize this observation, we tested for the MazF-mediated processing of the 16S rRNA indicated by the formation of the RNA43 by Ksg treatment [24]. To this end, total RNA was purified from strains MG1655 and MG1655Δ*mazF* grown in M9 minimal medium before and 60 min, 120 min, and 180 min after Ksg treatment or at the corresponding time points without addition of Ksg (Figure 1c,d). The formation of the RNA43 was followed by northern blot analysis using the probe V7 specific for the 3′-terminus of the 16S rRNA. The probe R25, specific for the 5S rRNA, was used as the loading control. In line with our previous results [24], growth in M9 minimal medium triggered the activation of MazF and consequently the generation of the RNA43 (Figure 5, lanes 1–4). Correspondingly, in the absence of the *mazF* gene RNA43 cannot be detected at time points 0 and 60 min (Figure 5, lanes 9–10). However, at the later time points 120 and 180 min we observed a faint signal (Figure 5, lanes 11–12). Intriguingly, we observed that Ksg treatment does not significantly stimulate the formation of RNA43 (Figure 5, lanes 5–8). In striking contrast to our expectations, Ksg treatment of strain MG1655Δ*mazF* likewise resulted in the generation of RNA43, though at a lower amount (Figure 5, lanes 13–16). Moreover, it is important to note that we observed a second signal upon Ksg treatment when employing probe V7, which indicates the presence of a smaller 3′-terminal 16S rRNA fragment (Figure 5, lanes 6–8 and 14–16).

## 3. Discussion

Selective protein synthesis in the presence of translational inhibitors is a well-known phenomenon [34,35]. However, the mechanisms allowing for the specialized translation are manifold and specific to the antibiotic used [34]. We have shown previously that treatment with the aminoglycoside antibiotic Ksg results in the formation of a ribosomal subpopulation that selectively translates lmRNA [15]. Direct binding of Ksg to the 30S ribosomal subunit induces the formation of 61S ribosomes, which lack several proteins of the small ribosomal subunit including proteins bS1, uS2, and bS21, all of which are required for translation of canonical mRNAs harboring a SD sequence [17,36]. Thus, the engendered ribosomes selectively translate lmRNAs [15]. Here, we show that in addition to the leaderless *cI-lacZ* reporter transcript several proteins are specifically synthesized during prolonged Ksg treatment. Given that the *mazEF* TA-system can be triggered by different antibiotics targeting the translational machinery [24,37] and active MazF leads to the formation of lmRNAs and RNAs with short leaders [24,25], this result prompted the hypothesis that the specific translation in the presence of Ksg could be attributed to activation of MazF. Thus, we further scrutinized the effect of the aminoglycoside antibiotic Ksg on the modulation of protein synthesis in *E. coli*.

Surprisingly, our data indicate that several of the selectively translated mRNAs are produced by alternative transcription at promoters close to the AUG start codon and only some transcripts are processed in a MazF-dependent manner. However, we cannot completely preclude the possibility that other endoribonucleases might contribute to the processing of mRNAs upstream of the AUG start codon, as we cannot identify the nature of the 5′-terminus by primer extension analysis. Nevertheless, our data reveal that besides RNA processing in response to Ksg treatment alternative transcription leads to the generation of transcripts with short leaders. Importantly, the common denominator of the stress response is the shortening of the 5′-UTRs of the respective mRNAs and the removal of secondary structures, as revealed by primer extension analysis on the *eno* mRNA (Figure 3g). However, our data reveal a disparity between the effect of Ksg, which only insignificantly triggers the MazF response, and other ribosome-targeting antibiotics like chloramphenicol and spectinomycin, which have been shown to strongly activate MazF, resulting in programmed cell death [21].

In complete contrast to our expectations, our results further indicate that treatment with the antibiotic Ksg leads to the removal of the 3′-terminal 43 nucleotides of the 16S rRNA and thus generation of RNA43 even in the absence of MazF (Figure 5). Moreover, a second shorter cleavage product is likewise generated in response to addition of Ksg. Together, these observations suggest that another endoribonuclease might be activated by the antibiotic, which could likewise target the rRNA at the same position. Given that the Ksg binding site is located adjacent to the cleavage site at nucleotide 1500 of the 16S rRNA [5,6] and that binding of Ksg to the ribosome results in the formation of protein-depleted ribosomes, we hypothesize that the structural rearrangement introduced by Ksg might allow for rRNA processing by other toxin endoribonucleases, which cannot access this position in the absence of Ksg due to sterical hindrance. Studies to identify the nature of the responsible mechanism resulting in the processing of the rRNA in the absence of MazF are currently ongoing.

### 3.1. Ribosome Heterogeneity Introduced by Kasugamycin

In this work we observed that the *rplL* mRNA coding for r-protein bL7/L12 as well as the *rimL* transcript encoding the bL12-serine acetyltransferase are selectively translated in the presence of Ksg. This result is intriguing given that the amount of protein bL12 was shown to be reduced whereas the amount of protein bL7 is increased under slow growth in minimal media, indicating that the majority of the protein is modified during adverse conditions [38]. This data corroborates our results obtained by 2D gel analysis performed upon pulse labeling of *E. coli* cells grown in the presence of Ksg (Figure 2). Under these conditions we only observed the synthesis of protein bL7, which is the acetylated form of bL12. Moreover, we verified the expression of the *rimL* gene encoding the bL12 modifying enzyme in the presence of Ksg (Table 1). These data suggest that in the presence of Ksg, the ribosome might be equipped predominantly with L7. As the ribosomal stalk complex composed of four copies of bL7/L12 is responsible for binding and recruiting translational factors [39] and acetylation of protein bL12 results in stabilization of the stalk [40], this modification could contribute to the modulation of ribosome activity and/or specificity underpinning the paradigm of ribosome heterogeneity as a means for fast and energy-saving reprogramming of the translational machinery in response to environmental changes [26,41,42].

## 4. Materials and Methods

### 4.1. Bacterial Strains, Plasmids, and Oligonucleotides

*E. coli* strains, plasmids, and oligonucleotides used in this study are summarized in Supplementary Tables S1 and S2, respectively. *E. coli* MG1655Δ*mazF* deletion strain was constructed by θP1 transduction [43] using the *E. coli* strain BW25113Δ*mazF* as donor (Keio Collection, [44]). The integrated kanamycin resistance cassette was removed by employing the Flp recombinase encoded by plasmid 706-Flp (Gene Bridges GmbH; Heidelberg, Germany) following the manufacturer’s instructions. Unless otherwise indicated, bacterial cultures were grown in M9 minimal medium [43]. Growth was monitored by measuring the optical density at 600 nm (OD_600_).

### 4.2. Pulse Labeling Experiments

*E. coli* cells (MG1655 and MG1655Δ*mazF*) harboring either plasmid pRB381-1 or pIM17 were grown at 37 °C in M9 medium (minimal medium, 10 μg/mL of each amino acid [43] without methionine. At an OD_600_ of 0.3, the cultures were divided and 0.5 mg/mL of Ksg was added to one half. Samples for pulse labeling were taken at indicated times before and after addition of Ksg or at the same time points from the cultures grown without Ksg. Then 0.2 mL of culture were withdrawn and pulse labeling was carried out by addition of 0.5 μL of [^35^S] methionine and incubation at 37 °C for 5 min. The reaction was stopped by addition of 100 μL cold methionine (20 mg/mL) and incubation at 37 °C for 5 min. Upon precipitation with 5% TCA the pellets were resuspended in 1× SDS-protein sample buffer, boiled for 5 min, and separated on a 12% SDS-PAGE to separate the proteins. For the different OD_600_ values, the same amount of total cellular proteins was subjected to electrophoresis. The gels were vacuum-dried and visualized using a Molecular Dynamics PhosphoImager (GE Healthcare, Buckinghamshire, UK).

### 4.3. Proteome Analysis

*E. coli* strain MG1655 harboring plasmid pRB381-1 was grown at 37 °C in M9 medium (minimal medium, 10 μg/mL of each amino acid [43] without methionine. At an OD_600_ of 0.3, the cultures were divided and 0.5 mg/mL of Ksg was added to one half. Five milliliters of culture were withdrawn 120 min after addition of Ksg and labeled with [^35^S] methionine, as described before. Total cellular protein extracts were analyzed by two-dimensional gel electrophoresis, as described before [45]. Equal amounts of cell material were dissolved in lysis buffer (8 M urea, 4%, *w*/*v*, CHAPS and 40 mM Tris-base) and the cells were disrupted by repeated freezing in liquid N_2_ and thawing at 37 °C. For the first dimension the Immobiline Dry strip pH 3 ± 10 (18 cm) (Amersham Pharmacia Biotech, Piscataway, NJ, USA) was used with the following IEF program: 12 h rehydration, 1 h 500 V, 1 h 1000 V, 4 h 8000 V (IPGphor isoelectric focusing system). Resolution in the second dimension was performed on 12.5% SDS polyacrylamide gels for 15 min at 10 mA and then for 5 h at 20 mA. Buffers and conditions were used according to the manufacturer’s instructions. The gels were dried and then exposed to a Molecular Dynamics Phosphor Imager screen and analyzed with PDQuest software (Bio-Rad, Hercules, CA, USA). For identification of selected proteins the respective spots were excised from the gel and the protein identities were assessed by mass spectrometry using a LTQ linear ion-trap (Thermo, Waltham, MA, USA) by the Mass Spectrometry Service Facility (Max F. Perutz Laboratories Support GmbH, VBC, Vienna, Austria).

### 4.4. Primer Extension Analysis

Primer extension analysis was performed exactly as described before [24]. The 5′-end-labeled reverse primers U49, K48, D17, and L44 specific for *rplL*, *clpP*, *cspA*, and *eno* mRNAs, respectively (Supplementary Table S2), were annealed to 10 μg of total RNA in 1× RT buffer by heating for 3 min to 80 °C, snap freezing in liquid nitrogen, and slowly thawing on ice. Primer extension reactions were performed in RT-buffer by using the AMV reverse transcriptase (Promega, Madison, WI, USA) by incubation at 42 °C for 15 min, essentially as described previously [14]. The generated cDNA samples were separated on an 8% polyacrylamid-8 M urea gel, and the extension signals were visualized using a Molecular Dynamics PhosphoImager.

### 4.5. Northern Blot Analysis

Total RNA was isolated using TRIzol^®^-reagent (Invitrogen, Carlsbad, CA, USA) following the manufacturer’s protocols. Three micrograms of purified RNA were fractionated on a 8 M UREA, 8% polyacrylamide gel, transferred to Hybond membrane (Amersham) using the Trans-Blot Semi-Dry Transfer Cell (Bio-Rad), and hybridized to [^32^P]-labeled oligonucleotides (Supplementary Table S2) exactly as described before to optimize for short RNA fragments [46]. The signals were visualized using a Typhoon PhosphorImager (Molecular Dynamics) and quantified with ImageJ software [47].

## Figures and Tables

**Table 1:** Selectively synthesized proteins in the presence of Ksg.

#	Gene	Protein Function	Length of 5′-UTR	MazF Processing in the 5′-UTR
1	*eno*	Enolase, enzyme of glycolysis, phosphoprotein, component of the RNA degradosome	76 nts	No
2	*rplL*	ribosomal protein L7/L12	*rplJ*-*rplL*-operon	Yes
3	*cspA*	cold-shock protein A	109 nts	No
4	*rplI*	ribosomal protein L9	*rpsF*-*rplI*-operon	No
5	*grcA*	stress-induced alternate pyruvate formate-lyase subunit	74 nts	Yes
6	*groES*	chaperone	72 nts	No
7	*uspA*	universal stress protein A	111 nts	No
8	*dnaK*	heat-shock chaperone	115 nts	No
9	*dnaJ*	heat-shock chaperone	*dnaK*-*dnaJ* operon	No
10	*rimL*	ribosomal-protein-L12-serine acetyltransferase	196 nts	No
11	*clpP*	Proteolytic subunit of ClpXP and ClpAP ATP-dependent proteases	32 nts	Yes
12	*groEL*	chaperone	409 nts	Yes
13	*rbfA*	ribosome binding factor required for processing of 16S rRNA	*infB*-operon	No

## Supplementary Materials

The following are available online at www.mdpi.com/2079-6382/5/2/19, Table S1: Bacterial strains and plasmids used in this study, Table S2: Oligonucleotides used in this study.
