# Supplementary material for: Insights into the Stress Response Triggered by Kasugamycin in Escherichia coli
Source: Antibiotics (Basel). 2016 Jun 1;5(2):19. doi: 10.3390/antibiotics5020019 (PMC4929434; doi:10.3390/antibiotics5020019)
Supplement: Supplementary file 1 [file antibiotics-05-00019-s001.pdf]

Supplementary Materials: Insights into the Stress Response Triggered by Kasugamycin Treatment in *E. coli*

Christian Müller, Lena Sokol, Martina Sauert, Oliver Vesper and Isabella Moll

Table S1. Bacterial strains and plasmids used in this study.

Strains and Plasmids	Relevant Features	Source or Reference
E. coli strains		
MG1655	F^{-}, lambda^{-}, *rph*-1	[1]
MG1655Δ*mazF*	MG1655, *mazF*$^{-}$	this study
BW25113Δ*mazF*	BW25113, *mazF*$^{-}$	[2]
Plasmids		
pRB391-1	pRB381 derivative harboring the first 63 codons of the λcI mRNA fused to the 8th codon of the lacZ gene	[3]
pIM17	pRB381 derivative harboring nucleotides −23–+69 of the *ompA* gene fused to the 8th codon of the lacZ gene	this study

Table S2. Oligonucleotides used in this study.

Name	Binding Region	Sequence
Oligonucleotides used for primer extension analysis		
U49	*rplL* from nt +67–+85	CTTCCATTGCAGAGATCAG
K48	*clpP* from nt +63–+80	CCGCGTGAGGTCTGTTCA
D17	*cspA* from nt +54–+80	GAGCCATCGTCAGGAGTGATGAAGCCG
L44	*eno* from nt +41–+56	GGGTTACCACGGGAG
Probes used for northern blot analyses		
V7	*rrsB* from nt 1541–1511	AAGGAGGTGATCCAACCGCAGGTTCCCCTACGGTTACC
R25	*rrfB* from nt 120–101	ATGCCTGGCAGTTCCCTACT

References

1. Blattner, F.R.; Plunkett, G., 3rd; Bloch, C.A.; Perna, N.T.; Burland, V.; Riley, M.; Collado-Vides, J.; Glasner, J.D.; Rode, C.K.; Mayhew, G.F.; *et al.* The complete genome sequence of *Escherichia coli* K-12. *Science* **1997**, *277*, 1453–1462.
2. Baba, T.; Ara, T.; Hasegawa, M.; Takai, Y.; Okumura, Y.; Baba, M.; Datsenko, K.A.; Tomita, M.; Wanner, B.L.; Mori, H. Construction of *Escherichia coli* k-12 in-frame, single-gene knockout mutants: The Keio collection. *Mol. Syst. Biol.* **2006**, *2*, doi:10.1038/msb4100050
3. Moll, I.; Huber, M.; Grill, S.; Sairafi, P.; Mueller, F.; Brimacombe, R.; Londei, P.; Blasi, U. Evidence against an Interaction between the mRNA downstream box and 16S rRNA in translation initiation. *J. Bacteriol.* **2001**, *183*, 3499–3505.
